# Prophylactic paclitaxel-eluting stent placement does not improve covered femoropopliteal stent patency^☆^

**DOI:** 10.1016/j.sopen.2021.09.004

**Published:** 2021-10-16

**Authors:** Rory Marples, Matthew Binks, Roberto Spina, Melissa Wright, Ravi Huilgol

**Affiliations:** a Wagga Wagga Rural Referral Hospital, Wagga Wagga, New South Wales; b University of New South Wales, Sydney, Australia; c St Vincent’s Hospital, Sydney; d University of Notre Dame, Australia

## Abstract

**Objective:**

Covered stents are an important tool in managing femoropopliteal peripheral arterial disease. However, their performance is impaired by edge neointimal hyperplasia and restenosis. We examined the effectiveness of prophylactic deployment of paclitaxel-eluting stents to prevent edge restenosis.

**Methods:**

A retrospective case–control study was performed. Patients with femoropopliteal peripheral arterial disease who were treated with Viabahn stent placement were compared to patients treated with Viabahn stents deployed in conjunction with paclitaxel-eluting stents (PTX). The primary outcome was time to loss of stent primary patency. The Kaplan–Meier method was used.

**Results:**

A total of 36 Viabahn and 25 Viabahn + paclitaxel-eluting stent procedures were evaluated, with mean follow-up periods of 27 and 18 months, respectively. The Viabahn + paclitaxel-eluting stent group had a longer length of vessel stented (P = .0023). Twelve-month primary patency was 74% in the Viabahn group and 75% in the Viabahn + paclitaxel-eluting stent group. Pre-existing dyslipidemia correlated with earlier loss of primary patency across the combined cohort (P = .0193).

**Conclusion:**

Viabahn stent primary patency is unaffected by the addition of paclitaxel-eluting stents.

## INTRODUCTION

Peripheral arterial disease (PAD) is a debilitating illness that afflicts almost 30% of people over the age of 70 years [1]. The superficial femoral artery (SFA) is the site most commonly affected by symptomatic disease [2]. Treatment options for SFA PAD and its extension into the popliteal artery (PA) include bypass surgery, angioplasty, and stenting. Offering reduced invasiveness, infection risk, and hospital stay, the latter 2 modalities have been widely adopted [3]. This is despite an increased risk of restenosis and occlusion when compared with bypass surgery [3,4]. Although carrying the risks of stent fracture and infection, stents have gained favor over angioplasty because of greater primary and secondary patency rates [5,6]. The greatest barrier to stent performance in managing SFA/PA occlusive disease is their susceptibility to neointimal hyperplasia (NIH) and restenosis [[7], [8], [9], [10]]. Stents coated with expanded polytetrafluoroethylene (ePTFE) have shown reduced levels of NIH and improved patency rates [7,[10], [11], [12]]. However, the performance of these stents has been hampered by the development of restenotic lesions at the proximal and distal edges of the devices [7,[11], [12], [13]]. Paclitaxel-eluting stents are recognized as effective treatments for SFA disease [14]. Initial research by Awwad et al (2015) reported that PTX stents could be used in conjunction with covered stents for long-segment occlusive SFA/PA lesions [15]. The interpretation of the results of this previous research is difficult due to the heterogeneous group of procedures reported, the lack of a control group, and the significant early occlusion rate observed.

We performed a retrospective case–control study to further investigate the method of prophylactic deployment of paclitaxel-eluting Zilver-PTX (PTX) (Cook Medical, Bloomington, IN) stents at the margins of Viabahn (VBN) stents (W.L. Gore and Associates, Flagstaff, AZ) to improve rates of edge restenosis and patency.

## METHODS

This study is a nonrandomized, retrospective study of consecutive patients treated by a single surgeon across 2 institutions. Ethics approval was granted from both institutions prior to data collection.

Demographic, comorbidity, and lesion characteristics were acquired for each patient by case note review, personal communication, and online image review (Table 1). *Stenotic PAD* was defined by an ultrasound peak systolic velocity ratio > 2.5 or angiographic evidence of > 50% stenosis. *Lesion length* was defined as the distance between the most proximal and distal stenoses of > 50% at index angiogram.

Patients were offered VBN therapy at the surgeon's discretion. This was based upon factors such as lesion length, presence of complete occlusion, and severity of calcification. Lesion inflow and outflow were optimized if required based on the judgement of the treating surgeon. Two patient cohorts were identified: (1) patients whose peripheral arterial disease was treated with the heparin-bonded Gore Viabahn stent only (VBN) and (2) patients who were treated with the combination of Viabahn stents and Zilver paclitaxel-eluting stents (VBN + PTX).

### Stenting Procedure

**Fig 1:**
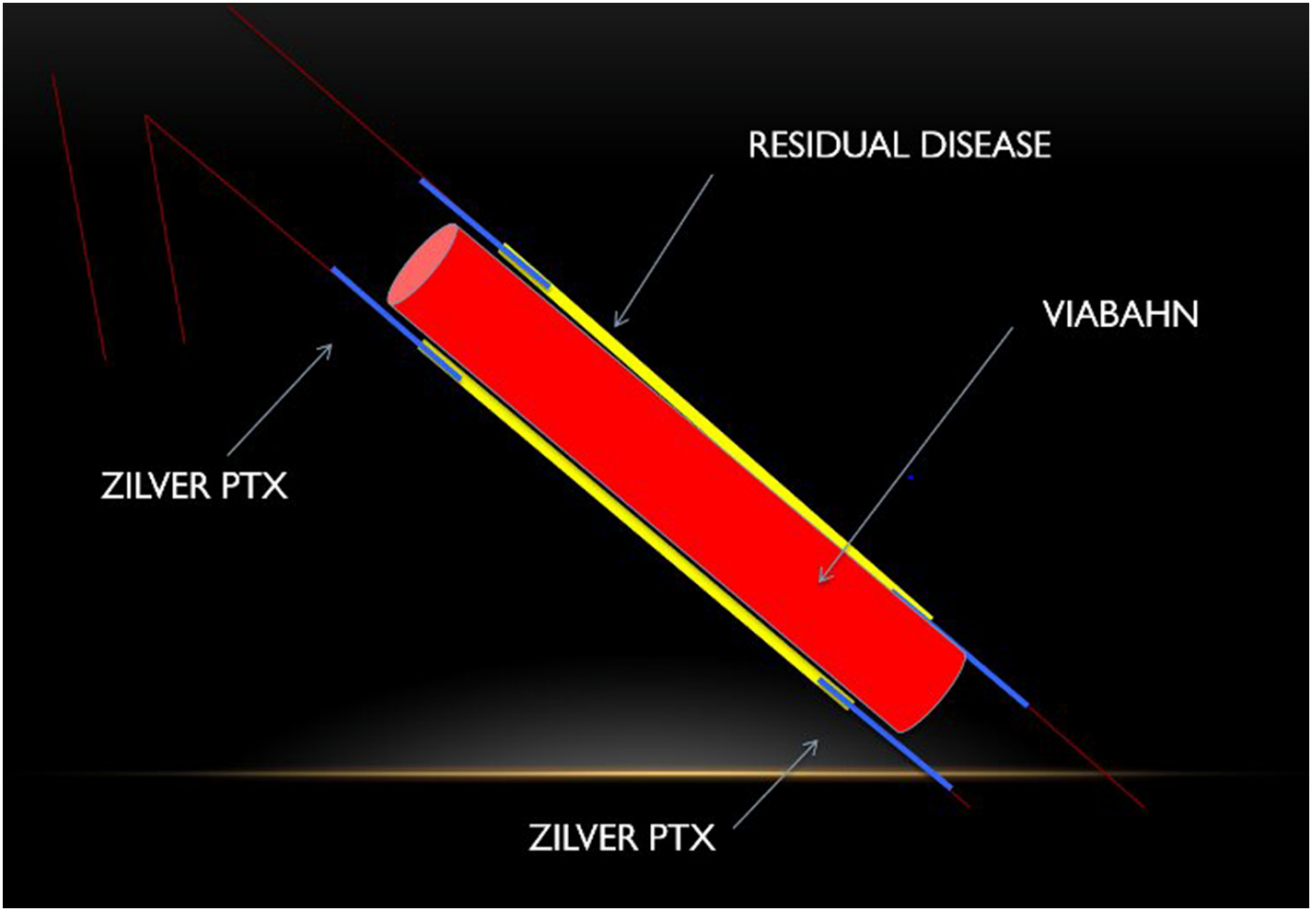
Image of procedural technique. The proximal and distal Zilver PTX stents are first deployed at the margins of the diseased segment. VBN stent(s) is(are) then deployed so as to line the intervening vessel lumen.

The lesion was assessed with angiography, and predilation was used if required. In the study group, Zilver PTX-eluting stents were deployed before VBN stent insertion (Fig 1), extending 10 mm from both the proximal and distal edge. The control group received the VBN stent only. Patients received dual antiplatelet therapy for 6 months postoperatively followed by a single agent thereafter. In patients taking an oral anticoagulant (eg, warfarin) preoperatively, this was continued postoperatively in combination with a single antiplatelet agent.

### Follow-Up

Patients were followed up with physical examination and duplex ultrasound at 6 weeks, 6 months, and 12 months postoperatively and yearly thereafter. Reintervention was performed at the discretion of the treating surgeon. Typically, this was for symptomatic restenosis or stenosis of > 75%. When asymptomatic restenosis with a range of 50%–75% was detected, this was followed up with more frequent ultrasonography.

### Study End Points

The study end point was time to loss of primary patency. *Loss of primary patency* was defined as either complete occlusion or a > 50% reduction of luminal diameter, as defined by a peak systolic velocity ratio > 2.5 with duplex ultrasound.

### Statistical Analysis

Time to loss of primary patency was estimated and compared between treatment groups using the Kaplan–Meier method. The relationships between specific covariates (demographic, lesion, and operative variables) and stent patency in the entire study cohort were examined using Kaplan–Meier curves.

## RESULTS

Sixty-one procedures were performed in total, 36 in the VBN group between 2011 and 2013 and 25 in the VBN + PTX group between 2012 and 2014. Mean follow-up in the VBN group was 27.25 months, and mean follow-up in the VBN + PTX group was 18.46 months.

Patient demographics were not significantly different between the 2 groups (Table 1). The VBN + PTX group had a statistically significant longer mean stented segment compared to the VBN group (37 vs 27.8 cm, *P =* .0023) (Table 1).

**Fig 2:**
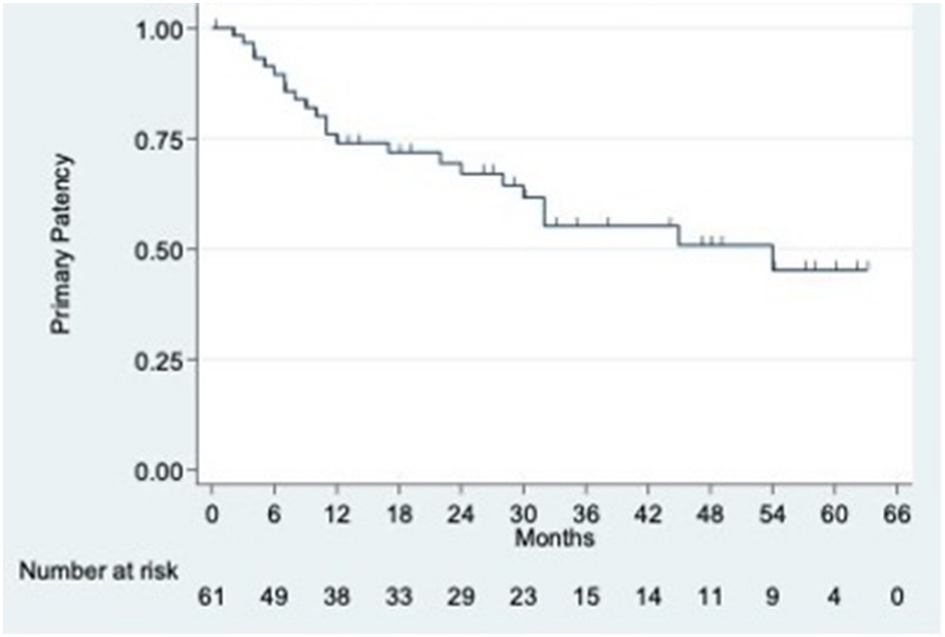
Kaplan–Meier-derived primary patency overall. Numbers below indicate limbs at risk at advancing study time points.

**Fig 3:**
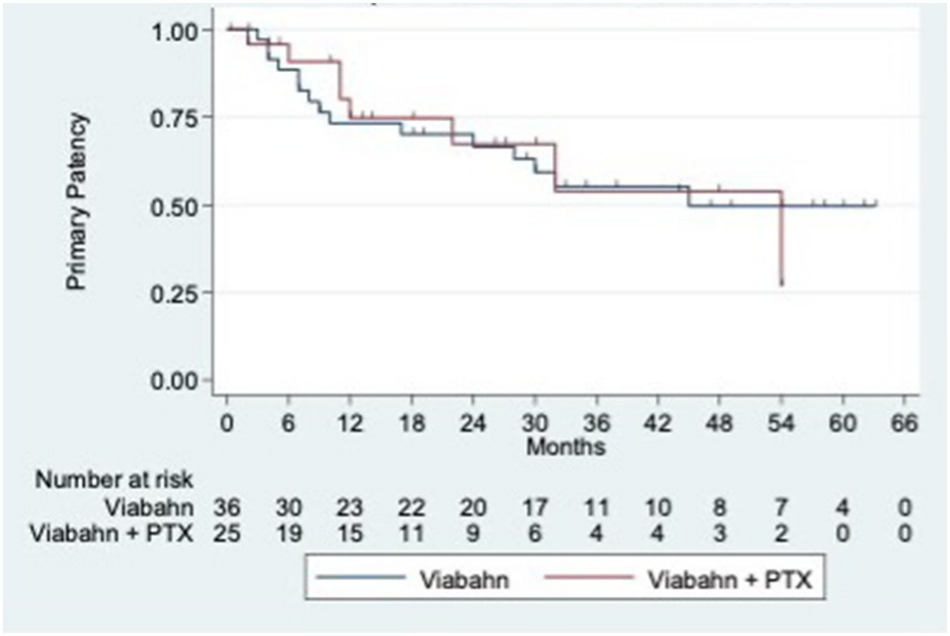
Kaplan–Meier-derived primary patency of VBN + PTX (red) versus VBN (blue) in SFA lesions of patients with symptomatic peripheral arterial disease.

Figure 2 shows the Kaplan–Meier curves for primary patency of the entire study cohort. Figure 3 compares the primary patency between the 2 groups using the Kaplan–Meier method. Twelve-month primary patencies were 74.0% overall, 73.2% in the VBN cohort and 74.5% in the VBN + PTX group. Twenty-four-month primary patencies were 67.0% overall, 66.6% in the VBN cohort and 67.3% in the VBN + PTX group.

**Fig 4:**
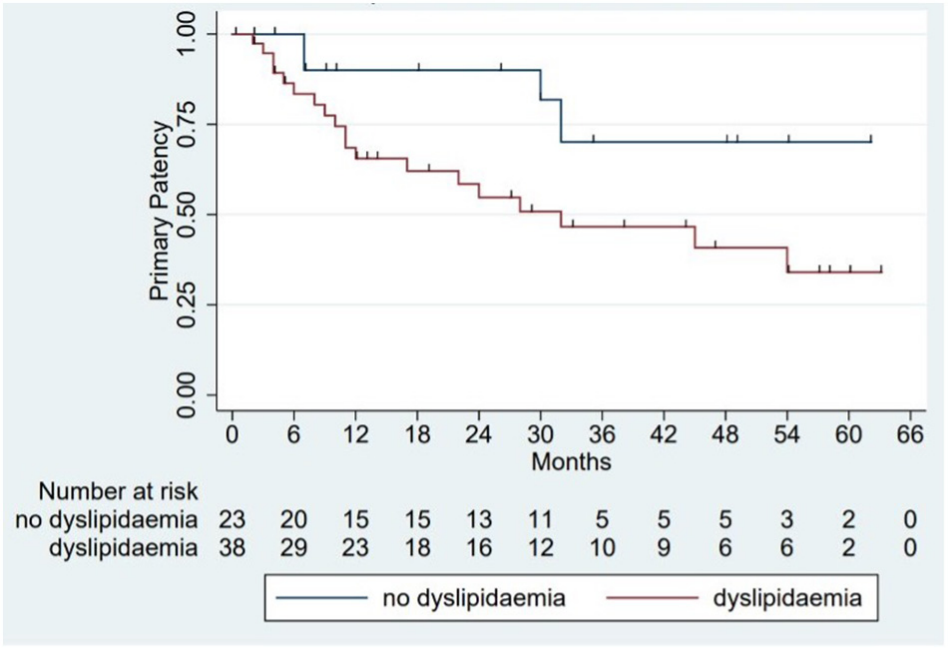
Kaplan–Meier survival curves of those patients with and without preexisting dyslipidemia. Red (1) = subjects with dyslipidemia.

Log-rank testing failed to reject the null hypothesis that when using VBN to treat SFA lesions, there is no difference in primary patency with the use of additional PTX stents (*P =* .9889). We also examined the relationship between specific covariates and the outcome variable (stent patency) with Kaplan–Meier curves. Univariate analysis of primary patency in the entire cohort demonstrated a statistically significant difference in time to loss of primary patency between patients with dyslipidemia and patients without dyslipidemia (*P =* .0193) (Fig 4). Primary patency was not affected by sex, smoking, diabetes, age (> 7 -years old), Rutherford class (stage 4 or greater), stented length, or number of runoff vessels.

Of the VBN group, 15 limbs (41.6%) required reintervention at a mean of 23.5 months postoperatively. Eight limbs (22.2%) experienced occlusive episodes at 3, 4, 5, 8, 10, 25, 28, and 41 months, respectively, and 7 (19.4%) restenosed at 7, 8, 10, 17, 24, 30, and 32 months. Of the occluded stents, 4 required surgical bypasses, 2 were relined with VBN stents, 1 was lysed before being relined with a PTX-eluting stent, and 1 was managed conservatively. Six of the restenoses were managed with angioplasty and PTX stenting (1 of which restenosed a second time and required bypass grafting), and the seventh was treated with a drug-coated balloon.

In the VBN + PTX group, 8 limbs (32%) required reintervention at a mean of 22.6 months postoperatively. Two limbs experienced occlusions at 8 and 15 months, respectively, and 6 restenosed at 6, 11, 11, 22, 32, and 54 months. One occlusion was managed with surgical bypass and the other with thrombolysis. All restenoses were treated with a combination of angioplasty and a further PTX-eluting stent. No amputations were required in either patient cohort during follow-up.

## DISCUSSION

Neointimal hyperplasia and edge restenosis reduce the efficacy of ePTFE-covered stents in managing femoropopliteal peripheral arterial disease. We examined the prophylactic deployment of paclitaxel-eluting stents at the edges of Viabahn covered stents to improve primary patency.

In comparing our overall results to the broader published literature, the 74.0% 12-month primary patency rate in our study is in keeping with previously reported results. The VIASTAR trial found a 71% 12-month primary patency rate in long lesions treated with VBN stents [16]. The VIPER trial demonstrated 12-month primary patency rates of 73% [12]. The 24-month primary patency of 67.0% in our study exceeds the > 50% benchmark recommended by the Society for Vascular Surgery practice guidelines [17].

Initially, the researchers looked to expand upon the research from Awwad et al by adding a control arm and using an alternate method of stent deployment to further evaluate the effectiveness of PTX in preventing edge restenosis. However, considering recent evidence, in the meta-analysis by Katsanos et al in 2018 that PTX-coated stents and balloons are associated with higher mortality rates, the researchers decided to revisit the data and assess whether PTX has any redeemable benefit in preventing edge restenosis, notwithstanding its association with higher mortality [18].

In their case series, Awwad et al (2015) reported results using a similar technique of combining drug-eluting stents with covered stents for the treatment of long-segment occlusive SFA/PA disease [15]. Our technique differs slightly by reversing the order of stent deployment, with PTX stent placement preceding that of VBN. We chose this order of stent placement to optimize drug-eluting stent contact with the endothelium. Our study concurs with the results of Awwad et al in that the addition of PTX stents to VBN stent placement is both feasible and safe. However, we add to the knowledge base of this technique by providing a direct comparison with a control group of VBN-only procedures, and our finding that the addition of PTX stents does not improve patency is an important guide to clinicians trying to optimize VBN stent patency. Furthermore, by examining this historical cohort in which PTX stenting was used prior to new evidence about its safety, we could retrospectively add to the conclusion of Katsanos et al that PTX is associated with mortality rates with evidence that it also provides no lasting benefit to the prevention of edge restenosis [18].

When examining the overall patency of both groups, of interest was the finding that dyslipidemia was associated with significantly worse primary patency. While we did not have the data to examine this in more detail, this finding provides prognostic information to clinicians treating femoropopliteal PAD with covered stents as well as provides further impetus for tight lipid control in these patients.

As would be expected, our study demonstrates that the VBN + PTX technique results in a greater length of stented vessel and more stents being required. The need for more stents increases the cost of the procedure with no perceived benefit to edge restenosis, as demonstrated in our study.

The limitations of this study include the lack of randomization and the small numbers in both study arms. The single operator in the study also limits external validity. Furthermore, the VBN + PTX cohort was treated later than the VBN cohort and consequently may have benefited from improved operator technique. The preference for solitary VBN deployment for short lesions during the early phases of PTX use may have favored this cohort. The small number of events beyond 24 months meant that small deviations in the survival curves were unlikely to be elicited.

In conclusion, we found that VBN stent primary patency is unaffected by the addition of PTX stents.

## Figures and Tables

**Table 1:** Demographic, lesion, and operative characteristics.

	*VBN*	*VBN + PTX*	P *value*
*N*	36	25	
Age (mean)	76yrs	78yrs	NS
Male	20/36 (55%)	10/25 (40%)	NS
Diabetes mellitus	9/36 (25%)	8/25 (32%)	NS
Hypertension	30/36 (83%)	22/25 (88%)	NS
Dyslipidemia	21/36 (58%)	17/25 (68%)	NS
Smoker	22/36 (61%)	13/25 (52%)	NS
Rutherford stage (mean)	3.3	3.4	NS
Complete occlusion	19 (53%)	18 (72%)	NS
Mean lesion length (cm)	25.47	30.36	NS
Stented segment length (mean)	27.8cm	37.0cm	.0023
Number of runoff vessels	2.25	2.2	NS
